# Population structure and microscale morphological differentiation in a freshwater snail from the Chilean Altiplano

**DOI:** 10.1186/s12862-023-02196-w

**Published:** 2024-01-06

**Authors:** Moisés A. Valladares, Alejandra A. Fabres, Fernanda Sánchez-Rodríguez, Gonzalo A. Collado, Marco A. Méndez

**Affiliations:** 1 Laboratorio de Biología Evolutiva, Departamento de Ecología, Facultad de Ciencias Biológicas, Pontificia Universidad Católica de Chile, Santiago, Chile; 2 Grupo de Biodiversidad y Cambio Global (GBCG), Departamento de Ciencias Básicas, Universidad del Bío-Bío, Chillán, Chile; 3 Laboratorio de Genética y Evolución, Departamento de Ciencias Ecológicas, Facultad de Ciencias, Universidad de Chile, Santiago, Chile; 4 Departamento de Ciencias Básicas, Universidad del Bío-Bío, Chillán, Chile; 5 Center of Applied Ecology and Sustainability (CAPES), Facultad de Ciencias Biológicas, Pontificia Universidad Católica de Chile, Santiago, Chile; 6 Institute of Ecology and Biodiversity (IEB), Faculty of Sciences, University of Chile, Universidad de Chile, Santiago, Chile; 7 Universidad de Magallanes, Puerto Williams, Chile

**Keywords:** Fragmentation, Aquatic invertebrate, South American summer monsoon, Geometric morphometrics, Pleistocene climatic oscillations

## Abstract

**Background:**

The diversity and population genetic structure of many species have been shaped by historical and contemporary climatic changes. For the species of the South American Altiplano, the historical climatic changes are mainly related to the wet events of great magnitude and regional influence that occurred during the Pleistocene climatic oscillations (PCOs). In contrast, contemporary climate changes are associated with events of lesser magnitude and local influence related to intensifications of the South American Summer Monsoon (SASM). Although multiple studies have analyzed the effect of PCOs on the genetic patterns of highland aquatic species, little is known about the impact of contemporary climate changes in recent evolutionary history. Therefore, in this study, we investigated the change in population structure and connectivity using nuclear and mitochondrial markers throughout the distribution range of *Heleobia ascotanensis*, a freshwater Cochliopidae endemic to the Ascotán Saltpan. In addition, using geometric morphometric analyses, we evaluated the concomitance of genetic divergence and morphological differentiation.

**Results:**

The mitochondrial sequence analysis results revealed the presence of highly divergent co-distributed and geographically nested haplotypes. This pattern reflects an extension in the distribution of groups that previously would have differentiated allopatrically. These changes in distribution would have covered the entire saltpan and would be associated with the large-scale wet events of the PCOs. On the other hand, the microsatellite results defined five spatially isolated populations, separated primarily by geographic barriers. Contemporary gene flow analyses suggest that post-PCO, climatic events that would have connected all populations did not occur. The morphometric analyses results indicate that there is significant morphological differentiation in the populations that are more isolated and that present the greatest genetic divergence.

**Conclusions:**

The contemporary population structure and morphological variation of *H. ascotanensis* mainly reflect the post-PCO climatic influence. Although both markers exhibit high genetic structuring, the microsatellite and morphology results show the preponderant influence of fragmentation in recent evolutionary history. The contemporary genetic pattern shows that in species that have limited dispersal capabilities, genetic discontinuities can appear rapidly, erasing signs of historical connectivity.

**Supplementary Information:**

The online version contains supplementary material available at 10.1186/s12862-023-02196-w.

## Background

The Altiplano landscape has experienced a complex environmental history influenced mainly by Andean orogenesis, glaciations and intense volcanic activity. During the Pleistocene climatic oscillations (PCOs), numerous drastic changes occurred in the temperature and rainfall of the region [1–3]. As a consequence of these changes, a series of humid and arid periods occurred in the Altiplano. The humid periods led to the formation of extensive paleolakes that connected currently isolated hydrological systems [4–6]. During these lake phases, multiple basins coalesced into paleolakes that would have connected, in extreme events, practically the entire Altiplano basin (e.g., the Titicaca-Desaguadero-Tauca system) during the Last Glacial Maximum [7, 8]. Later, during periods of aridity, fragmentation of the paleolakes would have occurred, giving rise to isolated hydrological systems we see today.

Various studies suggest that there is an association between the climatic changes that occurred during the Quaternary Period and the differentiation of the Altiplano taxa. Among the strictly aquatic animal models that support this hypothesis, research carried out on gastropods of the genus *Heleobia* Stimpson, 1865, and fish of the genus *Orestias* Valenciennes, 1839, stand out. In both cases, the results indicate that the species of these taxa would have diverged allopatrically during the transitions of wet and arid periods that occurred in the region [9–12]. On the other hand, the distribution patterns of co-distributed taxa, including gastropods of the genus *Biomphalaria* Preston, 1910, and amphipods of the genus *Hyalella* Smith, 1874, suggest that the evolutionary scenario of divergence of the highland biota could have involved multiple speciation processes (e.g., allopatric, secondary contact and dispersion) [13–17]. It is important to mention that while all the co-distributed taxa with *Heleobia* showed an influence of climatic changes in their evolutionary history, the differences in the patterns and processes are likely related to their life history traits. For example, *Biomphalaria* is a pulmonated gastropod, phylogenetically distant from *Heleobia*, and it exhibits physiological and phenotypic differences that are probably linked to the differences in the phylogeographic pattern observed between these species.

Although multiple studies have analyzed the effect of the PCOs on the diversification of the aquatic fauna of the Altiplano, few studies have focused on evaluating the effect of recent fragmentation (i.e., contemporary or post-PCO) on genetic divergence. This line of inquiry is particularly rare in macroinvertebrate groups that have historically received little attention and that in most cases lack taxonomic descriptions or systematic studies. On a recent timescale, the main environmental changes that have modified the landscape in the Altiplano are due to intensification events of the South American Summer Monsoon (SASM) [18–25]. These events have local effects and correspond to periods of increased in rainfall [26, 27] and, in extreme cases, produce catastrophic floods, such as those reported in 2015 in the Atacama Desert in Chile [28]. In this sense, although these events are not of the magnitude of the PCOs, it is likely that they have generated connections between nearby hydrological systems.

**Fig. 1:**
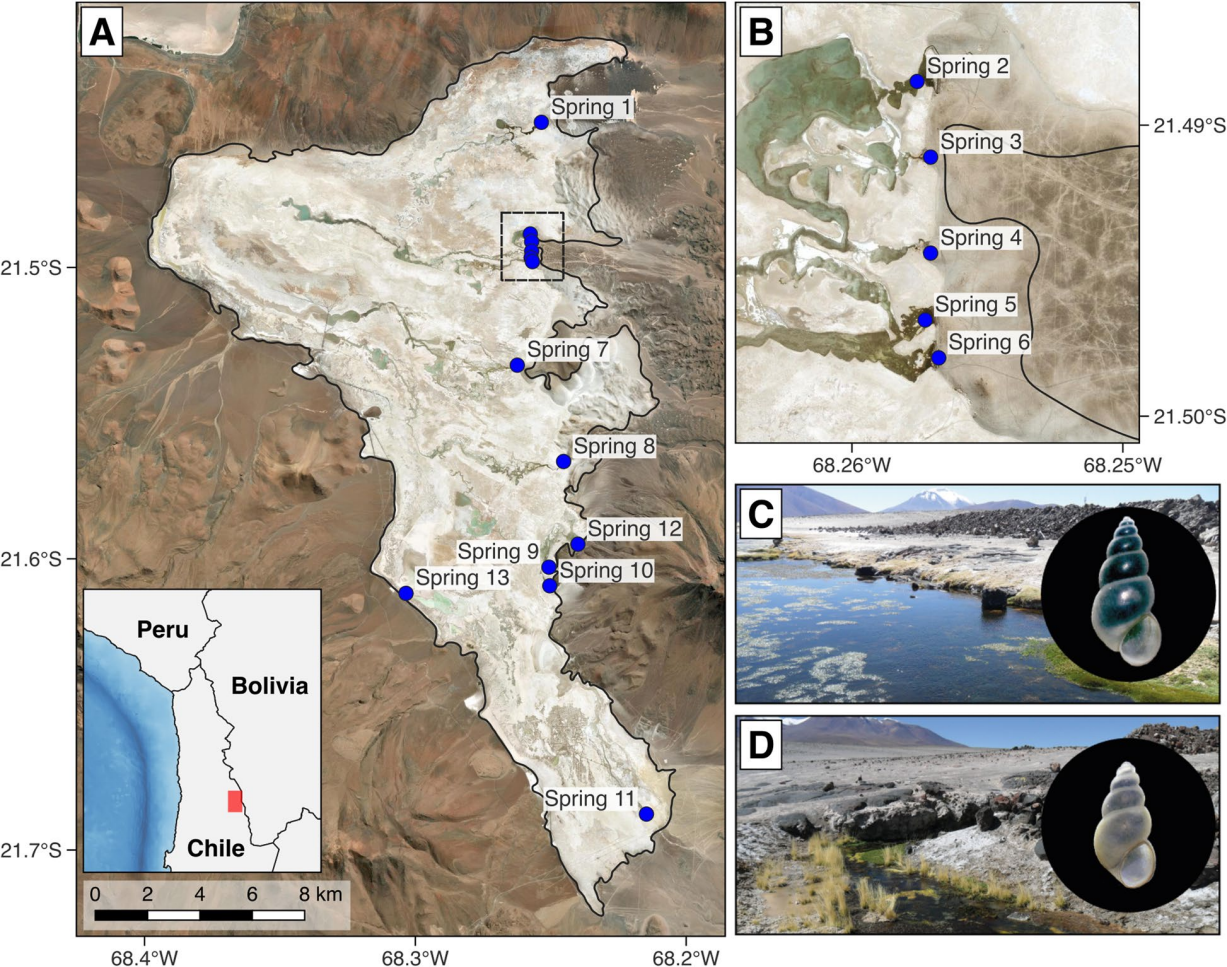
Sampling localities of *Heleobia ascotanensis* used in the present study. **A** Map showing the Ascotán Saltpan and the 13 springs analyzed. **B** Detail of springs 2 to 6. **C** and **D** correspond to photographs of springs 2 and 9, respectively. Each photograph shows representative individuals of each population (the black circle represents 5 mm). All photographs by Moisés A. Valladares. The map was made using QGIS Geographic Information System v3.4.9 with ESRI world imagery (ESRI, DigitalGlobe, GeoEye, Earthstar Geographics, CNES/Airbus DS, USDA, USGS, AeroGRID, IGN, and the GIS User Community) (http://www.qgis.org, accessed on April 10, 2023)

The Ascotán Saltpan (Antofagasta Region, Chile) is an endorheic basin of the southern Altiplano. The saltpan has a surface covered mainly by evaporite deposits, and the surface water is provided by a series of springs with different levels of connectivity (Fig. 1). For the strictly aquatic organisms of the saltpan, fragmentation of the habitat would have favored the differentiation of the populations, as terrestrial barriers prevented their dispersal and decreased the genetic flow between populations [14, 29–31]. Furthermore, in desert hydrological systems similar to the Ascotán Saltpan, it has been suggested that for freshwater gastropods, the isolation of the resident basins would induce considerable genetic differentiation [32]. Among the species that are distributed in the Ascotán Saltpan is *Heleobia ascotanensis* (Courty, 1907), and five subspecies that have been described based on conchological characters [33, 34]. *Heleobia ascotanensis* is a strictly aquatic snail; therefore, its distribution within the saltpan is restricted to the springs that make up the only bodies of freshwater within the system [30, 35]. It is important to mention that this restricted distribution to the springs of the saltpan could be modified, as passive dispersal mediated by birds may occur, as reported in similar freshwater snails from desert hydrological systems in Australia [36–38] or even over greater distances in the case of migratory birds [39]. However, there is no evidence that this bird-mediated dispersal occurs in *Heleobia*, and it has not been reported for other species from the Ascotán Saltpan or the Chilean Altiplano.

According to the environmental regulations in Chile, *H. ascotanensis* has been classified by the Ministry of the Environment as an endangered species (RCE DS38/2015), a classification based mainly on the decrease in the species’ habitat quality and since the species is estimated to occupy a range of less than 18 km^2^ [40]. According to the phylogenetic analysis carried out by Collado et al. [12] the populations of *H. ascotanensis* are genetically related to the highland populations of El Tatio (northern Chile) and Laguna Blanca (southern Bolivia); however, these relationships have not been evaluated in detail. Recently, a phylogeographic study of *H. ascotanensis* using the COI mitochondrial genetic marker suggested that three populations coexist in the system, which present low and moderate degrees of historical connectivity, in addition to exhibiting incipient morphological differentiation as a consequence of genetic and geographic isolation [30]. However, to date, no study has evaluated the structure and contemporary genetic flow of *H. ascotanensis* populations, for which more variable genetic markers are needed to describe the population structure and identify recent demographic processes (i.e., on a generational scale).

Based on the aforementioned background, the present study examined the genetic diversity, population structure and connectivity of *H. ascotanensis* populations using mitochondrial and nuclear DNA sequences (microsatellites). Considering the properties of each marker, this approach allowed us to contrast the historical population history obtained from the mitochondrial information with the contemporary history inferred from the microsatellite information. Additionally, the congruence between the genetic divergence and the degree of morphological differentiation among populations was evaluated using geometric morphometric analyses. By integrating multiple lines of evidence, we generated a detailed description of the evolutionary and demographic history of *H. ascotanensis*, illustrating how historical and contemporary habitat fragmentation processes have modulated population genetic diversity and promoted phenotypic differentiation in this species.

## Results

### Mitochondrial genetic structure and diversity

A total of 310 individuals from the 13 springs of the Ascotán Saltpan were sequenced for the COI gene. The alignment had a total length of 658 bp and presented 50 variable sites (24 were parsimony informative) that defined 56 haplotypes. Table 1 shows the values of the genetic diversity indices calculated from the COI sequences. The lowest genetic variability was found in Spring 9, where a single polymorphic site and two haplotypes were detected. In contrast, the greatest variability was found in Spring 13 (western margin of the saltpan), which presented 14 polymorphic sites and 16 haplotypes. On the other hand, Springs 2 to 6 presented the greatest intra-spring differentiation since, for all these systems, the average number of differences per pair of sequences was higher than 4.7, with a maximum of 5.437 in Spring 3.

**Table 1:** Genetic diversity indices obtained from COI gene sequences for each spring of the Ascotán Saltpan. The geographic coordinates for each spring, sample size (N), number of polymorphic sites (S), number of haplotypes (K), haplotype diversity (H) and average of differences between pairs of sequences (Π) are indicated

Site	Latitude (S)	Longitude (W)	N	S	K	H	Π
Spring 1	21.4501	68.2535	25	11	6	0.427	1.733
Spring 2	21.4885	68.2576	29	12	7	0.788	4.768
Spring 3	21.4911	68.2571	26	12	5	0.695	5.437
Spring 4	21.4944	68.2571	19	11	4	0.696	5.298
Spring 5	21.4967	68.2573	27	14	6	0.681	5.345
Spring 6	21.4980	68.2568	26	10	4	0.588	5.025
Spring 7	21.5336	68.2624	29	7	5	0.616	0.926
Spring 8	21.5667	68.2453	27	3	4	0.333	0.353
Spring 9	21.6029	68.2507	16	1	2	0.525	0.525
Spring 10	21.6093	68.2504	29	10	10	0.749	1.108
Spring 11	21.6877	68.2147	18	11	10	0.843	1.634
Spring 12	21.5950	68.2400	13	4	5	0.692	0.974
Spring 13	21.6119	68.3035	26	14	16	0.954	3.480
**Total**			**310**	**50**	**56**	**0.837**	**4.574**

**Fig. 2:**
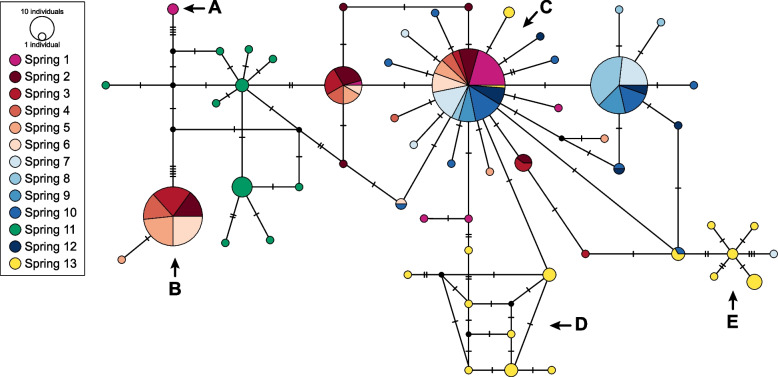
Haplotype network of *Heleobia ascotanensis* from the Ascotán Saltpan generated using COI gene sequences. The segments on the lines represent the number of mutational steps. The size of the circumferences represents the relative frequency of each haplotype. **A**, **B**, **D** and **E** indicate divergent haplotypes, and **C** indicates the higher-frequency haplotype (details in main text)

In the haplotype network (Fig. 2), it was observed that within the saltpan, there were no clearly differentiated haplogroups, and only Spring 11 presented solely exclusive haplotypes. On the other hand, a divergent haplotype was detected in Spring 1 (separated by five mutational steps from the rest of the network, A in the figure), and a high-frequency haplotype was present only between Springs 2 to 6 (separated by six mutational steps from the rest of the network, B in the figure). In general, the network showed that many of the haplotypes are shared between springs, with the exception of those that occur in Spring 11. The grouping of the haplotypes of Springs 11 and 13 shows a dispersed and extended network. On the other hand, for the rest of the springs, the grouping pattern exhibits star-shaped branching. The network shows a central haplotype (C in the figure) that encompasses all springs, except for Spring 11. Spring 13 included multiple exclusive haplotypes (D and E in the figure) and representatives of the main haplotype (C in the figure).

The genetic differences between pairs of springs of the Ascotán Saltpan obtained from the COI gene are shown in Table 2 (below the diagonal). The results indicate that Spring 11 is the most differentiated system since it presents the highest *F*_ST_ values, and all comparisons were significant. Spring 13 (western margin of the saltpan) also shows high differentiation values. In general, the results of the *F*_ST_ test suggest that genetic divergence is associated with the spatial positions of the springs, since more distant springs presented higher differentiation values.

**Table 2:** *F*_ST_ values between pairs of springs of the Ascotán Saltpan. The distances calculated using the mitochondrial genetic sequences of the COI marker are shown below the diagonal, and the microsatellite results are shown above the diagonal. For the COI marker, the significant results (*p* < 0.05) are highlighted in bold. For microsatellites, the analysis was performed using the ENA correction for null alleles and 10,000 bootstrap replicates

	S1	S2	S3	S4	S5	S6	S7	S8	S9	S10	S11	S12	S13
**S1**		0.047	0.038	0.082	0.050	0.046	0.073	0.118	0.113	0.101	0.120	0.112	0.153
**S2**	**0.185**		0.024	0.149	0.050	0.101	0.127	0.171	0.148	0.132	0.174	0.144	0.195
**S3**	**0.360**	0.029		0.068	0.003	0.038	0.083	0.119	0.111	0.110	0.121	0.111	0.196
**S4**	**0.352**	0.006	0		0.055	0.019	0.092	0.125	0.135	0.152	0.093	0.153	0.248
**S5**	**0.407**	0.068	0	0		0.028	0.071	0.103	0.105	0.112	0.112	0.111	0.194
**S6**	**0.439**	0.088	0	0	0		0.061	0.096	0.104	0.121	0.078	0.135	0.211
**S7**	**0.157**	**0.325**	**0.485**	**0.492**	**0.522**	**0.553**		0.012	0.037	0.051	0.055	0.051	0.128
**S8**	**0.453**	**0.429**	**0.551**	**0.572**	**0.582**	**0.612**	**0.218**		0.036	0.067	0.058	0.059	0.142
**S9**	**0.211**	**0.305**	**0.450**	**0.456**	**0.488**	**0.520**	0	**0.176**		0.023	0.093	0.030	0.139
**S10**	**0.120**	**0.309**	**0.472**	**0.476**	**0.510**	**0.540**	0	**0.249**	0.000		0.098	0.040	0.085
**S11**	**0.620**	**0.346**	**0.378**	**0.392**	**0.409**	**0.442**	**0.742**	**0.826**	**0.763**	**0.724**		0.123	0.144
**S12**	**0.102**	**0.256**	**0.407**	**0.405**	**0.448**	**0.480**	0	**0.307**	0	0	**0.714**		0.143
**S13**	**0.238**	**0.302**	**0.412**	**0.401**	**0.447**	**0.470**	**0.314**	**0.463**	**0.317**	**0.297**	**0.550**	**0.240**	

The Mantel test was used to analyze the significance of the correlation between the matrix of geographic distances and genetic distances (*F*_ST_) obtained using COI sequences; a Pearson’s correlation coefficient *r* = 0.33 (*p* < 0.05) was obtained. This result suggests that there is a significant relationship between geographic distance and genetic differentiation. Therefore, the pattern of genetic divergence of the *Heleobia* populations of the Ascotán Saltpan is congruent with a scenario of isolation by distance.

### Nuclear microsatellite markers

Genotypes were obtained from 390 individuals from the 13 springs of the Ascotán Saltpan (10 microsatellite loci). This set of data contained all the individuals of the mitochondrial analyses; in addition, the sample size was increased in all springs. All the loci (except the locus Hel45) showed deviations from Hardy-Weinberg equilibrium in at least one of the springs (Table S1). The diversity indices showed that eight of the 13 springs presented private or exclusive alleles (Table 3); in this sense, Springs 8 and 13 showed the highest number of private alleles (A_P_ = 5) in the saltpan. In 10 of the 13 springs, a deficit of heterozygotes (H_O_ < H_E_) was detected, which was confirmed by high values of the fixation index (F). Similarly, in 11 springs, positive values of internal relatedness (i.e., inbreeding) were detected; in particular, the values were higher for individuals of Springs 4, 6 and 8. Only individuals of Springs 10 and 12 presented IR values close to zero (i.e., unrelated parents).

**Table 3:** Genetic diversity indices of *Heleobia ascotanensis* populations determined using 10 microsatellite loci. The number of samples (N), number of alleles (A), number of deprived alleles (A_P_), average allelic richness (A_R_), percentage of polymorphic loci (% _PL_), observed heterozygosity (H_O_), expected heterozygosity (H_E_), fixation index (F) and internal relatedness (IR) are shown. For % _PL_, H_O_, H_E_, F and IR, the population mean of the parameter and its standard error are shown

Site	N	To	A_P_	A_R_	% P_L_	H_O_	H_E_	F	IR
Spring 1	30	53	1	2.20	100	0.52 ± 0.08	0.54 ± 0.06	0.01 ± 0.11	0.20 ± 0.04
Spring 2	30	40	0	2.02	100	0.50 ± 0.09	0.48 ± 0.06	−0.04 ± 0.12	0.21 ± 0.04
Spring 3	30	55	0	2.16	100	0.48 ± 0.07	0.53 ± 0.06	0.08 ± 0.10	0.29 ± 0.05
Spring 4	30	43	4	1.99	80	0.26 ± 0.08	0.43 ± 0.09	0.46 ± 0.12	0.47 ± 0.06
Spring 5	30	43	1	2.07	100	0.43 ± 0.08	0.50 ± 0.06	0.12 ± 0.13	0.27 ± 0.07
Spring 6	30	44	3	2.09	100	0.35 ± 0.07	0.48 ± 0.08	0.26 ± 0.10	0.42 ± 0.07
Spring 7	30	52	0	2.32	100	0.46 ± 0.07	0.59 ± 0.04	0.21 ± 0.10	0.35 ± 0.05
Spring 8	30	50	5	2.18	100	0.38 ± 0.08	0.53 ± 0.07	0.26 ± 0.12	0.39 ± 0.05
Spring 9	30	57	0	2.33	100	0.48 ± 0.06	0.59 ± 0.04	0.19 ± 0.10	0.27 ± 0.05
Spring 10	30	60	0	2.40	100	0.64 ± 0.07	0.62 ± 0.04	− 0.02 ± 0.09	0.03 ± 0.04
Spring 11	30	53	2	2.23	90	0.41 ± 0.09	0.53 ± 0.08	0.20 ± 0.13	0.32 ± 0.04
Spring 12	30	61	3	2.36	100	0.60 ± 0.06	0.62 ± 0.04	−0.01 ± 0.12	0.09 ± 0.04
Spring 13	30	69	5	2.25	100	0.60 ± 0.09	0.54 ± 0.08	−0.10 ± 0.10	0.15 ± 0.03
**Total**	390	124							
Mean ± SE					97.7 ± 1.7	0.47 ± 0.02	0.54 ± 0.02	0.12 ± 0.03	0.27 ± 0.01

The paired *F*_ST(SSR)_ test results (Table 2, above the diagonal) show that in general, the geographically close springs presented low differentiation values. This phenomenon was detected, for example, among Springs 1 to 7 (except for some comparisons of Spring 2) and between Springs 8 and 12. Spring 13 presented the highest differentiation values with respect to the rest of the systems, except for the comparison with Spring 10. The Mantel test between the matrix of geographic distances and microsatellite genetic distances (*F*_ST(SSR)_) yielded a Pearson’s correlation coefficient *r* = 0.54 (*p* < 0.05). The correlation coefficient was higher than that obtained using mitochondrial data, confirming the importance of isolation and geographic distance in the genetic structure of *H. ascotanensis*.

### Population genetic structure

**Fig. 3:**
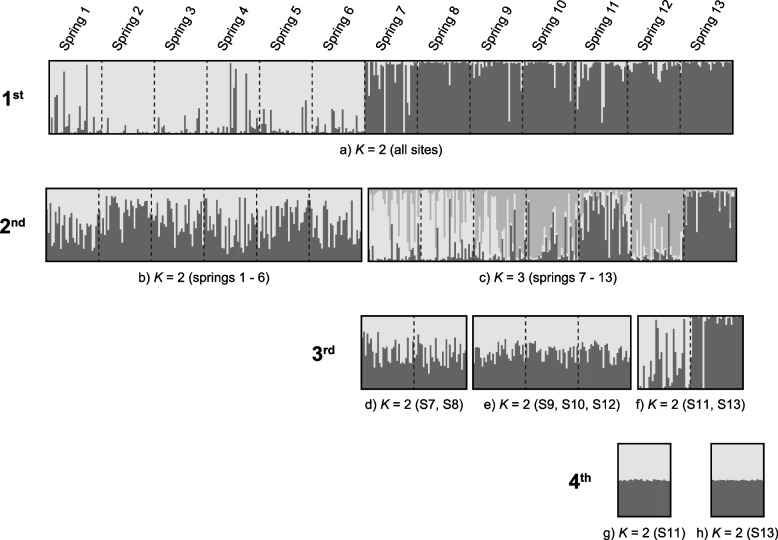
Population structure of *Heleobia ascotanensis* from 10 microsatellite loci using a hierarchical Structure analysis

The most likely number of populations in the initial Structure round, determined using Evanno’s method, was K_SSR_ = 2 (Fig. 3). This partitioning of the dataset clearly segregated the northern and southern springs of the Ascotán Saltpan. A second round of Structure analysis was performed for each of the groups separately. In the second round of the northern group, no obvious structuring was detected; therefore, Springs 1 to 6 constituted a population (hereinafter Population 1). On the other hand, the second round of the southern group defined three populations (K_SSR_ = 3), and this new partitioning of the data also reflected the spatial arrangement of the springs in the saltpan. In the third round of the southern group, the population formed by Springs 7 and 8 (Population 2) and the population composed of Springs 9, 10 and 12 (Population 3) were defined. Finally, in the fourth round of Structure, the most isolated springs of the saltpan were analyzed, and both were defined as independent populations (Spring 11 constituted Population 4, and Spring 13 Population 5).

**Fig. 4:**
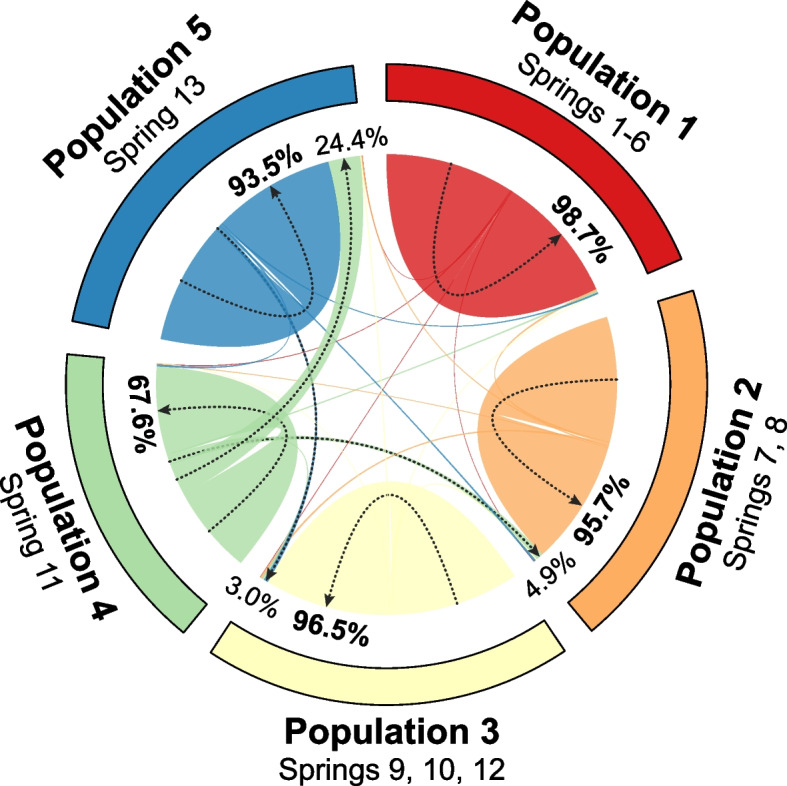
Estimated migration rates for *Heleobia ascotanensis* populations from 10 microsatellite loci in the Ascotán Saltpan. The groupings correspond to the populations defined by Structure. Rates greater than 2% are indicated, and the self-recruitment rate is highlighted in bold

The migration rates estimated using BayesAss showed high self-recruitment values for Populations 1, 2 and 3 (98.7, 95.7 and 96.5%, respectively) (Fig. 4). In addition, none of these populations was a source of migration to another population with values greater than 1% (Table S2). With respect to the most isolated populations, Population 4 (Spring 11) presented the lowest self-recruitment value in the saltpan, at 67.6%, and contributed 24.4% to Population 5 (Spring 13) and 4.9% to Population 2 (Springs 7 and 8). Finally, Population 5 presented a moderate rate of self-recruitment (93.5%) and contributed 3.0% of migrants to Population 3 (Springs 9, 10 and 12).

No significant signs of genetic bottlenecks were detected in any of the populations defined by Structure using both the two-phase model (TPM) and the stepwise mutational model (SMM) in Bottleneck (all *p* values > 0.1 in the Wilcoxon tests). Similarly, the five populations presented normal L-shaped distributions, indicating the absence of abrupt demographic declines in the populations.

### Geometric morphometric analyses

**Fig. 5:**
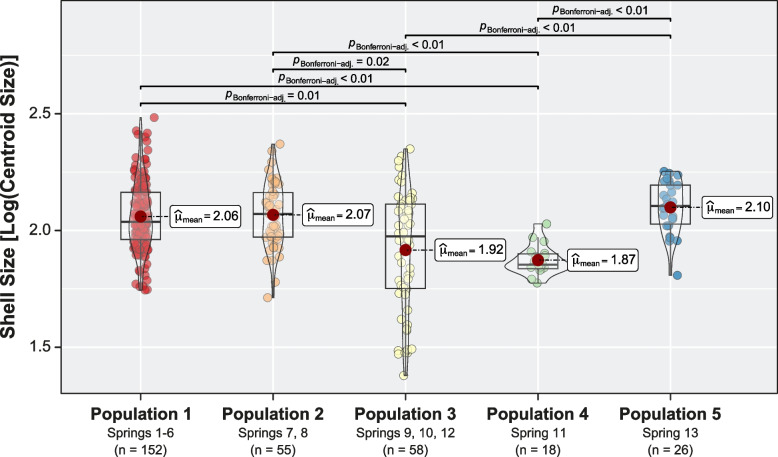
Morphological differentiation associated with shell size among *Heleobia ascotanensis* populations. Differences of the logarithm of the size of the centroid classifying the individuals according to the populations defined by Structure. The population mean of the centroid size is shown, and significant differences are indicated (Bonferroni post hoc)

Significant differences in the size distributions (response variable) considering the populations defined by Structure were evaluated by means of a Procrustes ANOVA. In all analyses, the logarithm of the centroid size [Log (Centroid Size)] was used as a proxy for the size of the specimens. The results showed significant differences in the size distribution (*F* = 13.81; *Z* = 5.60; *p* < 0.05) between populations. Post hoc comparisons indicated that Populations 3 and 4 (Springs 9, 10 and 12 and Spring 11, respectively) presented significant differences in the size of the centroid compared to the rest of the populations (Fig. 5).

**Fig. 6:**
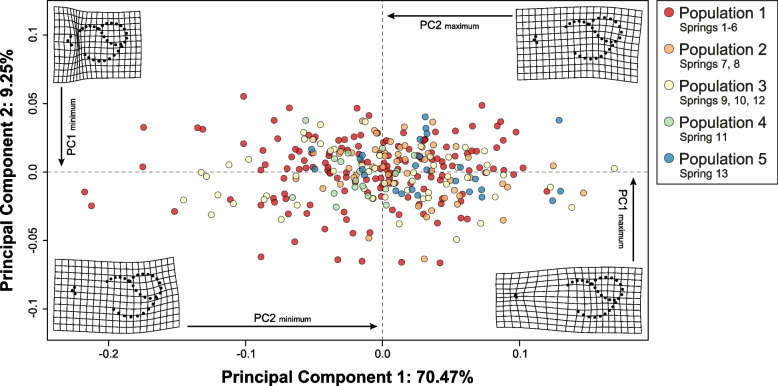
Morphological space of the populations of *Heleobia ascotanensis*. Principal component analysis using the GPA matrix of the populations defined by Structure. The deformation grids for the extreme shapes of each axis of variation in relation to the global consensus are shown in the corners of the graph

The principal component analysis of the individuals classified by population showed an overlap in the morphological space (Fig. 6), and only Population 4 (Spring 11) encompassed a bounded region in the center of the morphospace. The deformation grids for extreme values of Principal Component 1 (PC1) showed that this axis was mainly associated with the size and elongation of the shell. On the other hand, PC2 was associated with the position and shape of the peristome and showed the differentiation in the relative width of the shell of the populations of *H. ascotanensis*. The MANOVA results showed significant differences between populations (*F* = 2.08; *Z* = 12.56; *p* < 0.05). The penalized discriminant analysis (PDA) for the individuals classified by population had an overall precision (for the entire dataset) of 90% (*p* < 0.05). The percentages of correct classification by population were Population 1 = 95%; Population 2 = 87%; Population 3 = 74%; Population 4 = 89%; and Population 5 = 96%.

## Discussion

In the present study, genetic diversity was estimated from mitochondrial and nuclear data, and the demographic history and population structure of the gastropod *Heleobia ascotanensis* from the Ascotán Saltpan were inferred. The results indicate that the analyzed samples are structured into five populations that present different degrees of diversity, differentiation and genetic flow. This pattern is expected considering that hydrological systems composed of springs with a certain degree of isolation are habitats where endemic species have evolved in a variety of groups, which apparently have species in relatively stable and isolated conditions [35, 41–44]. On the other hand, the morphometric analyses results indicate that there is incipient morphological differentiation in the populations that are more isolated. Furthermore, although there is considerable overlap in the multivariate space, the analyses revealed significant differences in all comparisons. The results obtained using mitochondrial sequences are consistent with those reported by Valladares et al. [30]. In both cases, high genetic structuring was detected among the *Heleobia* populations. However, differences were also detected in the composition of the populations, and this situation is probably related to the incorporation of a new locality (Spring 13).

The genetic pattern obtained from microsatellite loci was partially discordant with that obtained from mitochondrial data. In this sense, although both sources of evidence suggest a marked phylogeographic structure influenced by the geographic isolation between the springs, the microsatellite data reflect a more prevalent modulation of the landscape. A result that supports this proposition is that for both sets of data, a significant and positive relationship was detected between the geographic distance of the springs and the genetic distance of the populations. This correlation is consistent with signals of a pattern associated with isolation by distance. However, the Pearson correlation index of the Mantel tests was higher for microsatellites (*r*_microsatellites_ = 0.54) than for mitochondrial sequences (*r*_COI_ = 0.33). It is likely that this discordance is due to the rapid rate of variation of microsatellite markers compared to that of mitochondrial sequences [45, 46]. Therefore, these markers are much more sensitive to the contemporary geographic and environmental influence imposed by the system [47].

The mitochondrial sequence analysis results showed great genetic variability and high intra-spring differentiation in Springs 2 to 6 (north of the saltpan). In the haplotype network, two haplotypes separated by multiple mutational steps (B and C in Fig. 2) were detected in these springs, a finding accompanied by high values of differences between pairs of sequences (Π). According to Valladares et al. [30], this pattern reflects an allopatric divergence between groups (i.e.*,* they come from different more recent common ancestors, MRCA), followed by an extension in the distribution range of the C haplotype from the south of the saltpan to Springs 2–6. Considering the great geographic extension of haplotype C, it is probable that the extension of the range of this species occurred during the climatic events of great magnitude that characterized the PCO and that connected the more isolated springs (Springs 1 and 13). This hypothesis is congruent with our results and is also consistent with the results of phylogeographic studies carried out in the endemic fish *Orestias ascotanensis* Parenti, 1984, of the Ascotán Saltpan [29, 31]. In contrast, the microsatellite results did not show evident genetic segregation among individuals from the northern springs of the saltpan (Population 1, Springs 1–6). However, the inferred ancestry ratios in Structure suggest that the individuals came from two ancestral populations. On the one hand, this result supports the hypothesis that two genetically divergent groups co-occurred in the northern springs of the saltpan and later intermixed. On the other hand, unlike the mitochondrial pattern, the microsatellite results suggest that recently (i.e.*,* post-PCO), climatic events of great magnitude that connected all the springs have not occurred. In contrast, habitat fragmentation would be the predominant condition, and population dynamics would mainly reflect the influence of geographic isolation.

Gene flow analysis using microsatellite markers suggests that migration between populations occurs asymmetrically and in a unidirectional direction from south to north. In this sense, Population 1 (north of the saltpan, Springs 1–6) would be the most isolated since it showed the highest rate of self-recruitment (98.7%) and would not receive migrants from other populations. Populations 2 and 3 (center of the saltpan) also showed high rates of self-recruitment and would also receive migrants from Populations 4 and 5, respectively. Although Population 5 (Spring 13) is located on the western margin of the saltpan and a great distance from the rest of the systems, the results show that it would receive a large proportion of migrants from Population 4 (Spring 11). Finally, Population 4 is the southernmost in the saltpan and presented the lowest self-recruitment rate (67.6%). This unidirectional flow from south to north can be associated with the altitudinal profile of the saltpan since the southern springs are higher than those located in the northern part of the system [29, 48]. Therefore, in events of flooding of the saltpan, a product of anomalies in the periods of precipitation [27], runoff would occur mainly from the south to the north. This hypothesis is compatible with the connectivity models that have been proposed for the Ascotán Saltpan and that have been evaluated in different aquatic organisms [14, 29–31].

The morphological analysis results showed that there are significant differences in the size and shape of the shell between the populations of *H. ascotanensis*. The geometric morphometrics approach was used to detect subtle differences in the shell and is a valuable tool that could be used in systematic studies. This approach is important considering that in the shell of *Heleobia,* there are no meristic features such as spines, ribs or other morphological characters that can be used as diagnostic features in the identification or delimitation of species. Although all populations showed significant differences in shell shape, Population 4 (Spring 11) also showed differences in size. This result is important because molecular analyses consistently show genetic differentiation between individuals in this population and the rest of the system. Considering both sources of evidence (morphology and genetics), the divergence process is more pronounced in Population 4 with respect to the rest of the populations of the species. According to Valladares et al. [30], the population of Spring 11 would have been isolated from the rest of the system during the last three lake cycles that occurred in the Altiplano (Sajsi [c. 21–25 kya], Tauca [c. 14–18 kya] and Coipasa [c. 11–13 kya]). Therefore, it is likely that the temporal and geographic isolation in which Population 4 has evolved has led to morphological differentiation and genetic divergence. A similar pattern was detected in Population 5 (Spring 13; western margin of the saltpan); however, the morphological differentiation was less than that detected in Population 4 and presented haplotypes shared with other springs.

The results of the present study show that it is necessary to resort to different sources of evidence to obtain a robust understanding of the pattern of population structuring. The latter is of particular importance when the species of interest is distributed in a complex hydrological system of springs with different degrees of connectivity. Our results and studies carried out in similar desert systems [29–31, 44, 47, 49–52] show that defining populations a priori based on geographical aspects is not appropriate. Recognizing this precaution is crucial in the case of threatened species such as *H. ascotanensis* since the correct description of the population structure and evolutionary history are necessary to undertake adequate conservation strategies [53–55]. In this sense, although genetic bottlenecks were not observed in any of the populations, the elevated levels of inbreeding in the majority of springs raise concerns. This pattern is probably modulated by geographic isolation, as shown by the high values of internal relatedness of the springs that make up the most isolated populations. However, this pattern is likely due to the intrinsic components related to the low vagility and reproductive strategy (direct development) reported for the species of the genus *Heleobia* [47, 56]. Our results contribute to the knowledge of the species and fauna of macroinvertebrates of the South American Altiplano. Future studies should integrate more variable markers (e.g., SNPs) to decipher the finer genetic pattern but should also undertake monitoring that assesses temporary changes in the population structure.

## Conclusions

Multiple complementary methods were used to evaluate the genetic diversity, population structure, demographic history and morphological differentiation of *Heleobia ascotanensis*. The results of the analysis of the mitochondrial sequences suggest that the historical genetic structure would have been influenced mainly by the wet events associated with the PCO that would have connected currently isolated springs. In contrast, the contemporary population structure inferred using microsatellite markers reflects the fragmentation of the system due to the arid conditions that have prevailed in the post-PCO Altiplano. Five populations were defined that were consistent with the geography of the saltpan and the degree of isolation of the springs. The results of the gene flow analysis showed that migration occurs only from south to north, responding to the altitudinal profile of the saltpan. Finally, the morphometrics study showed that genetic differentiation was accompanied by significant differences in the shape and size of the shell.

## Methods

### Study and sampling area

Individuals of *Heleobia ascotanensis* were collected from 13 springs of the Ascotán Saltpan (Antofagasta Region, Chile) in 2019. The sampling was authorized by the Undersecretary of Fisheries and Aquaculture (SUBPESCA) in accordance with Exempt Resolution No. 2604/19. The sampling sites corresponded to 12 springs of the eastern margin of the saltpan (Springs 1 to 12, S1–S12) and a spring located in the western margin of the system (Spring 13, S13) (Fig. 1). The individuals used for the molecular analyses were previously photographed to be included in the subsequent morphological analyses. To avoid problems associated with allometric changes during ontogeny, the morphological study used adult individuals that were in the upper third of the size distribution of each sample collected.

### Extraction and amplification of DNA sequences

The DNA samples were extracted and amplified by PCR following the protocol of Valladares et al. [30, 47]. For the COI marker, both strands of the amplification product were sequenced by Macrogen Inc. (Seoul, Korea). The nucleotide sequences were edited in the CodonCode Aligner v.8.0.1 program (CodonCode Corporation, Dedham, MA, USA) and were then aligned using the MAFFT v7.505 algorithm using the web server [57, 58].

For microsatellite markers, seven specific primers were developed for *H. ascotanensis* following the protocol of Fabres et al. [59]*,* and three other primers previously reported for the congeneric species *Heleobia atacamensis* (Philippi, 1860) were used [59]. The amplification products were analyzed by the sequencing service of the Pontificia Universidad Católica de Chile (Santiago, Chile). Then, fragment analysis was performed using GeneMapper v4.0 software (Applied Biosystems, Waltham, MA). Subsequently, the excess of homozygotes or heterozygotes was checked with Micro-Checker v2.2.3 [60], and for each locus, the presence of stutters and null alleles were searched. The details of the mitochondrial and microsatellites are indicated in Table S3.

### Mitochondrial DNA sequence analysis

The genetic diversity of each spring was described by the following indices calculated in DnaSP v6.0.1 [61]: number of polymorphic sites (S), number of haplotypes (K), haplotype diversity (H) and average number of differences between pairs of sequences (Π). The relationships between the haplotypes recovered for the COI gene were visualized by constructing a haplotype network using the median-joining algorithm [62] in PopART v1.7 [63]. To examine the differentiation between each pair of systems, a paired *F*_ST_ test was performed in the Arlequin v3.5 program [64]. The significance of each paired *F*_ST_ index was obtained based on 10,000 permutations. The existence of a pattern of genetic divergence associated with a phenomenon of isolation by distance (IBD) was tested by means of a Mantel test using matrices of genetic distances (dependent variable) and geographic distances (independent variable). The spatial distance matrix corresponded to the geographic distance in meters standardized between the systems studied. The genetic distance matrix was linearized according to the formula [*F*_ST_/(1- *F*_ST_)] proposed by Rousset [65]. The analysis was performed through 10,000 permutations using the vegan package v2.5–6 [66] in R software v3.6.1 [67].

### Analysis of microsatellite sequences

Possible deviations from Hardy-Weinberg equilibrium were evaluated by loci and populations using an exact test in Genepop v4.7.5 [68]. Indices of genetic diversity considering the number of alleles (A), private number of alleles (A_P_), percentage of polymorphic loci (% P_L_), observed heterozygosity (H_O_) and expected heterozygosity (H_E_) and fixation index (F) were calculated in GenAlEx v6.5 [69]. Allelic richness (A_R_) was calculated in the PopGenReport v3.0.4 package [70] using rarefaction considering the population with the smallest sample size. To estimate the degree of inbreeding, the population mean internal relatedness (IR) was calculated in the Rhh v1.0.2 package [71]. The IR index assigns positive or negative values, where positive values suggest inbred individuals and negative values suggest exogamous individuals, while values close to zero suggest that the individuals come from unrelated parents [72]. Finally, a paired matrix of *F*_ST(SSR)_ (where SSR denotes microsatellite results) was calculated in FreeNA v1.0 [73] (using the null allele exclusion correction, ENA), which was subsequently used to evaluate the existence of a pattern of genetic divergence associated with an IBD phenomenon following the same procedure used for mitochondrial sequences.

The number of genetic groups (K_SSR_) was evaluated in Structure v2.3.4 [74–76] by performing multiple rounds of analysis using a hierarchical approach [77]. The decision to apply a hierarchical approach was based on the evolutionary history of *H. ascotanensis*. Previous studies have identified two genetically differentiated groups in the saltpan and proposed the occurrence of secondary contact between these groups [12, 30, 35]. This background suggests the presence of two groups with substantial genetic differentiation that could potentially mask the detection of additional populations. Therefore, given that Structure “accurately detects the uppermost hierarchical level of structure” [78], we opted to employ a hierarchical approach. In the first round, the complete dataset was analyzed to estimate the number of groups considering all the springs of the saltpan. Subsequently, the process was repeated recursively for each group detected in the previous round. The analysis was repeated until no population structure was detected in the estimated groups. In all rounds, a Bayesian analysis was performed using the MCMC algorithm that considered 600,000 iterations and a burn-in of 300,000 parameters. In addition, the results were obtained based on 15 runs. The structuring parameters were calculated for each K value using the admixture model and allele frequencies correlated between groups [74, 75]. The most likely number of populations (K_SSR_) was evaluated considering the value of the log likelihood of the observed data (LnP [D]) and the rate of second-order change in the logarithm of the data in different runs of *K* (ΔK) described in Evanno et al. [78]. ΔK was calculated using the Structure Harvester v0.6.94 online platform [79]. The results were summarized and compared in Clumpak v1.1 [80].

To estimate the contemporary gene flow between populations defined by Structure, a Bayesian analysis was performed using BayesAss v3.0.8 software [81]. This analysis was performed using 50 million iterations and a 30% burn-in period, with sampling every 5000 iterations. The mixing parameters for allelic frequencies, migration rates and inbreeding coefficients were defined as 0.30, 0.10 and 0.30, respectively. Ten independent runs were performed to examine the consistency in the results, and the convergence of each run was visualized with an MCMC trace plot. The Bayesian deviation measure was used to determine the run that showed the best fit of the model [82, 83].

Recent bottlenecks for the *H. ascotanensis* populations defined by Structure were estimated using Bottleneck v1.2 [84], based on the graphical analysis of Luikart et al. [85] and by Wilcoxon signed-rank tests (WSRs). Considering the nature of the dataset, the two-phase model (TPM) and the stepwise mutational model (SMM) were used to simulate the mutation-drift equilibria. For TPM, a two-phase mutation model was defined with 95% stepwise mutations, 5% multistep mutations and a variance (*σ*^2^_g_) of 12.

### Geometric morphometric analyses

The external morphology of the individuals of *H. ascotanensis* was analyzed using geometric morphometrics, which allows an empirical comparison of the complex shapes presented by the shells. In *Heleobia,* a morphological change associated with sexual dimorphism has not been detected [12, 30, 56], so males and females were included in the analyses. The shell of each specimen was photographed from the ventral view using a digital camera (Moticam 5) coupled to a stereomicroscope (Motic SMZ-168). For the geometric morphometric analyses, the 310 individuals of the mitochondrial genetic study (COI) were analyzed, except for one individual from Spring 7 that presented damage to the peristome. The morphological study of the shell was carried out by classifying the individuals according to the populations defined by Structure.

Morphological analyses were carried out considering eight landmarks and 26 semi landmarks located in the extension of the entire shell. The landmarks were positioned at the apex of the shell and at the sutures present in the first, second and last turn of the shell. The semi landmarks were placed equidistant on the curves that connected the landmarks of the last turn of the shell and on the peristome (Fig. S1). The position of the landmarks was established to capture the main characteristics of the shape of the shell and functional traits associated with locomotion and feeding (opening of the shell). The location of the morphological landmarks was determined in the StereoMorph v1.6.7 package [86]. Using the geomorph v4.0.4 package [87], the landmark coordinates were aligned using a generalized Procrustes analysis (GPA). To determine the morphological differentiation associated with size, the GPA matrix (shell shape information) and the size of the log-transformed centroid (shell size information) were jointly analyzed. The shell morphospace was explored by principal component analysis (PCA) using the values of the morphological covariance matrix. After checking the assumption of data normality using the Shapiro-Wilk test in R, to quantify the degree of morphological variation associated with shell size between populations, a Procrustes ANOVA was performed on the morphological covariance matrix and the size of the centroid. The Bonferroni correction was applied to adjust the *p*-values of the multiple comparisons. To evaluate the variation in the shell shape between populations, a MANOVA was performed on the morphological covariance matrix. For the analyses, statistical significance was estimated through permutations (Residual Randomization in Permutation Procedures, RRPP; 10,000 iterations) [88–90] and a Pillai-Bartlett distribution was used. Finally, to determine whether individuals could be correctly assigned to each population using the shell shape (covariance matrix), a linear discriminant function analysis (LDA) was performed in R. The accuracy of the LDA classification was evaluated through a 10-fold cross-validation method, where the dataset is divided into 10 equally sized groups. Nine groups are utilized for training the model, while one group is set aside for validation.

### Supplementary Information

**Additional file 1: Table S1.** Characteristics of the 10 microsatellites used in the study and significant deviations of Hardy-Weinberg Equilibrium by populations after Bonferroni corrections (*p* < 0.05). **Table S2.** Estimated contemporary migration rate for *Heleobia* populations of the Ascotán Saltpan obtained using 10 microsatellite loci. Standard deviation is shown in parentheses. The self-recruitment rate is indicated along the diagonal. **Table S3.** List of primers sequences used in the study for the mitochondrial and microsatellites markers. **Fig. S1.** Geometric morphometrics in *Heleobia ascotanensis*. The landmarks and semilandmarks used in the morphological study are presented.

**Additional file 2: Table S1.** Microsatellite allele data for 390 individuals genotyped at 10 loci.

## Data Availability

Sequences generated in this study have been deposited in GenBank under the Accession numbers OQ847095–OQ847404.
