# Supplementary material for: Population structure and microscale morphological differentiation in a freshwater snail from the Chilean Altiplano
Source: BMC Ecol Evol. 2024 Jan 6;24:5. doi: 10.1186/s12862-023-02196-w (PMC10770964; doi:10.1186/s12862-023-02196-w)
Supplement: Supplementary file 1 — Additional file 1: Table S1. Characteristics of the 10 microsatellites used in the study and significant deviations of Hardy-Weinberg Equilibrium by populations after Bonferroni corrections (p < 0.05). Table S2. Estimated contemporary migration rate for Heleobia populations of the Ascotán Saltpan obtained using 10 microsatellite loci. Standard deviation is shown in parentheses. The self-recruitment rate is indicated along the diagonal. Table S3. List of primers sequences used in the study for the mitochondrial and microsatellites markers. Fig. S1. Geometric morphometrics in Heleobia ascotanensis. The landmarks and semilandmarks used in the morphological study are presented. [file 12862_2023_2196_MOESM1_ESM.pdf]

Supplementary Material

Population structure and microscale morphological differentiation in a freshwater snail from the Chilean Altiplano

Moisés A. Valladares[1,2], Alejandra A. Fabres[3], Fernanda Sánchez-Rodríguez[3], Gonzalo A. Collado[2,4] and Marco A. Méndez[3,5,6]

[1] Laboratorio de Biología Evolutiva, Departamento de Ecología, Facultad de Ciencias Biológicas, Pontificia Universidad Católica de Chile, Santiago, Chile

[2] Grupo de Biodiversidad y Cambio Global (GBCG), Departamento de Ciencias Básicas, Universidad del Bío-Bío, Chillán, Chile

[3] Laboratorio de Genética y Evolución, Departamento de Ciencias Ecológicas, Facultad de Ciencias, Universidad de Chile, Santiago, Chile

[4] Departamento de Ciencias Básicas, Universidad del Bío-Bío, Chillán, Chile

[5] Centro de Ecología Aplicada y Sustentabilidad (CAPES), Facultad de Ciencias Biológicas, Pontificia Universidad Católica de Chile, Santiago, Chile

[6] Instituto de Ecología y Biodiversidad (IEB), Facultad de Ciencias, Universidad de Chile, Santiago, Chile

Supplementary Table 1. Characteristics of the 10 microsatellites used in the study and significant deviations of Hardy-Weinberg Equilibrium by populations after Bonferroni corrections ($p < 0.05$).

Locus	Motif	Size range	Significant Hardy-Weinberg deviations
TAT_01	$(GTCC)_{10}$	190–218	S1, S2, S4, S7, S8, S9
TAT_02	$(TCTG)_{10}$	250–298	S4, S11
TAT_04	$(TTCT)_{16}$	190–270	S3, S4, S6, S7, S9, S10, S11, S12
TAT_14	$(TAT)_{7}$	252–282	S3, S5, S6, S8, S9, S11, S12
TAT_17	$(GTC)_{9}$	87–231	S1, S2, S3, S4, S5, S6, S8, S10, S12, S13
TAT_24	$(AG)_{7}$	70–102	S1, S3, S4, S6, S7, S8, S13
TAT_27	$(CG)_{10}$	320–354	S1, S2, S5, S8, S9, S13
Hel_45	$(CA)_{6}$	240–242	--
Hel_32	$(CA)_{13}$	206–232	S11
Hel_09	$(GAT)_{6}$	186–243	S1, S2, S3, S4, S5, S6, S7, S8, S9, S10, S11, S12

Supplementary Table 2. Estimated contemporary migration rate for *Heleobia* populations of the Ascotán Saltpan obtained using 10 microsatellite loci. Standard deviation is shown in parentheses. The self-recruitment rate is indicated along the diagonal.

From/To	Population 1	Population 2	Population 3	Population 4	Population 5
Population 1	0.9869 (0.0057)	0.0026 (0.0025)	0.0033 (0.0031)	0.0047 (0.0038)	0.0026 (0.0025)
Population 2	0.0147 (0.0103)	0.9574 (0.0182)	0.0142 (0.0127)	0.0052 (0.0051)	0.0085 (0.0080)
Population 3	0.0056 (0.0053)	0.0172 (0.0122)	0.9651 (0.0148)	0.0035 (0.0035)	0.0085 (0.0071)
Population 4	0.0115 (0.0112)	0.0485 (0.0274)	0.0193 (0.0156)	0.6763 (0.0094)	0.2444 (0.0315)
Population 5	0.0096 (0.0092)	0.0132 (0.0123)	0.0300 (0.0188)	0.0121 (0.0112)	0.9350 (0.0243)

Supplementary Table 3. List of primers sequences used in the study for the mitochondrial and microsatellites markers.

Marker	ID/Locus	Ta (°C)	Primer sequence	Reference
SSR	TAT_01	58	F: GACATAGTAGCTAGTACCTTG	This study
			R: GAGTGTAATGTGTACGTGAT	
	TAT_02	58	F: TACTGTAGTGACCTTACTGTG	
			R: GACAGACAGAGGACAGATA	
	TAT_04	58	F: TCGTTAGAGTACAGTCTTGT	
			R: GACTGTTCTTGATATGCTAC	
	TAT_14	56	F: GATGTAGTCTATGTCCTAGCT	
			R: ACACAGACATGCACACTA	
	TAT_17	56	F: GTTGATAGTCTAGTGTCAGTG	
			R: TCTAAGGTACCCATGTATC	
	TAT_24	58	F: GGATGAGTACAGTAGCTACTA	
			R: AGTCTGAGTCTGAGTCTGAG	
	TAT_27	56	F: GGTAGAACACACAAGTAAAC	
			R: CAAGTACACACAAGATCAGT	
	Hel_45	56	F: AAGAACATGTCTCCTACAG	Fabres et al., 2020
			R: GTCTGTGACTTAGATTATGG	
	Hel_32	58	F: CTTCAGGAAGTTGATAGAC	
			R: CTGTGTTTAAAGAGAGTGTC	
	Hel_09	56	F: CGTACTTCTATGAGAATCTG	
			R: TCATCATAGTCATCACTGTC	
mtDNA	COI		F: LCO1490	Folmer et al., 1994
			R: HCO2198	

Note: Ta is the annealing temperature.

Supplementary Figure 1. Geometric morphometrics in *Heleobia ascotanensis*. The landmarks and semilandmarks used in the morphological study are presented.

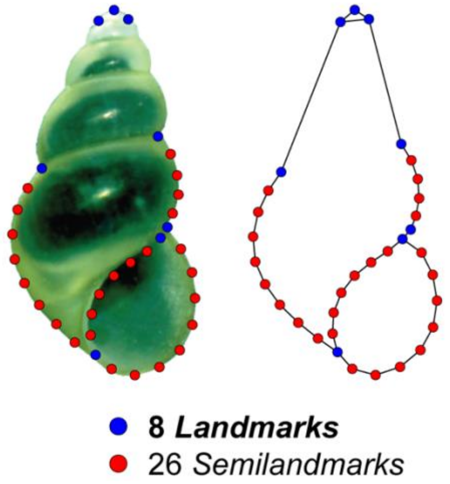

References

Folmer, O., Black, M., Hoeh, W., Lutz, R. & Vrijenhoek, R. DNA primers for amplification of mitochondrial cytochrome c oxidase subunit I from diverse metazoan invertebrates. Mol Mar Biol Biotech 3, 294–9 (1994).

Fabres, A. A. et al. Novel microsatellite markers for an endangered freshwater snail, *Heleobia atacamensis* (Caenogastropoda: Cochliopidae), from the Atacama Saltpan. Molluscan Res 40, 231–235 (2020).
